# Legume breeding for intercropping is indispensable for species expected to face high competition

**DOI:** 10.3389/fpls.2026.1838691

**Published:** 2026-06-29

**Authors:** Paolo Annicchiarico, Elisa Calastri

**Affiliations:** Research Centre for Animal Production and Aquaculture, Council for Agricultural Research and Economics (CREA), Lodi, Italy

**Keywords:** agroecology, competitive ability, genetic correlation, genetic gain, grain legume, mixing ability, perennial legume, plant-plant interaction

## Abstract

Legume-based intercrops exploit plant functional diversity to raise and/or stabilize crop yields and quality while enhancing ecosystem services. The need for specific legume breeding for intercropping depends crucially on the genetic correlation (*r_G_*) for genotype yields across pure stand (PS) and target mixed stand (MS) conditions. This review aimed to assess the size of *r_G_* as a function of competitive stress exerted on the legume and discuss its implications along with complementary information supporting cost-efficient breeding strategies. Following a literature review, we retained 13 data sets of perennial legumes, warm-season or cool-season grain legumes including at least 14 genotypes (lines, cultivars, etc.) evaluated in MS and PS. Their pooled information revealed a significant (*P* < 0.001) linear decrease of *r_G_* with the increase of competitive stress that explained 67% of *r_G_* variation. The predicted efficiency of indirect selection in PS relative to direct selection in MS averaged 0.61, with wide variation (0.42-1.07) mainly determined by *r_G_* variation (0.40-1.00). These results and other indications suggested that specific breeding for intercropping is indispensable for legume species expectedly facing high competition (as determined by associated species/cultivars and crop management), which seemingly are the majority. In this situation, large-scale legume selection could rely on genotype yield in MS with a few (possibly mixed) highly productive non-legume testers, since greater legume competitive ability could also improve the total yield of intercrops with likely modest negative impact on indirect genetic effects leading to higher yield of non-legume companions, whereas specific mixing ability effects are expectedly small.

## Introduction

The simultaneous cultivation of legume and non-legume species is a long-standing diversification practice that was already adopted over four thousand years ago in regions of the Old or New World such as Egypt (Malleson, 2016) and Central America (Mota-Cruz et al., 2025). With the rise of modern agriculture, intercropping remained confined to subsistence agriculture (Altieri, 2004) and, in temperate regions, perennial forage production (especially white clover-grass mixtures) (Frame and Newbould, 1986). A new interest for legume-based intercropping has arisen in the prospect of an agroecological transition of modern agriculture that exploits plant functional diversity for a sustainable intensification of crop production (Brooker et al., 2015; Justes et al., 2021). In particular, intercropping could raise and/or stabilize crop yields and crop quality (Kiær et al., 2009; Bedoussac et al., 2015; Yu et al., 2015; Li et al., 2023), while simultaneously enhancing ecosystem services and reducing adverse environmental impacts (Tamburini et al., 2020; Wang et al., 2025). The potential advantages of intercropping are particularly important for organic and low-input farming systems (Bedoussac et al., 2015; Jensen et al., 2015; Schneider et al., 2015) but benefits may be substantial also in other systems (Yu et al., 2015).

The expansion of intercropping requires the availability of cultivars capable of producing mixtures that combine high total yield with a balanced proportion of their component species (Bourke et al., 2021; Moore et al., 2022). These characteristics ought to be verified by suitable cultivar registration systems (Hohmann et al., 2026). Modern cultivars are usually selected and evaluated under pure stand (PS) conditions. Crucial questions for breeders are whether to breed grain or forage legumes specifically for intercropping and how to do that cost-efficiently, also in view of the many possible companion cultivars and the currently modest seed market size for this type of cropping system. Cultivar adaptation to intercropping may depend on the competitive ability under specific growing conditions, temporal complementarity of growth pattern, complementarity of aerial or root growing patterns, similarity of crop maturity, and other characteristics of the pair of associated cultivars of the two species (Litrico and Violle, 2015; Annicchiarico et al., 2019; Demie et al., 2022; Kiær et al., 2022; Moore et al., 2022). Contrasting lines of evidence support either the selection under mixed stand (MS) (e.g., Sharma et al., 1993; Annicchiarico, 2003) or the view that selecting under pure stand (PS) could also produce cultivars well adapted to intercropping (e.g., Padi, 2007). On the whole, long-standing work on legume-grass perennial forages and more recent work on grain legume-cereal intercrops indicate that the performance of genotypes (intended hereafter as inbred lines, clones, cultivars, families, populations, or hybrids) across PS and MS conditions may be largely inconsistent, especially for legume species that tend to be outcompeted under the adopted MS conditions of nitrogen fertilization level, sowing densities, and vigor of the associated species and cultivar (Annicchiarico et al., 2019; Brooker et al., 2024). From a quantitative genetics perspective, breeding program decisions on selection in PS or MS environments depend crucially on the extent of the genetic correlation for genotype responses of the focal species across PS and the target conditions of MS (Annicchiarico et al., 2019; Dubey et al., 2024). This parameter summarizes the portion of genotype × environment interaction across PS and MS environments that is relevant for breeding (Falconer, 1952). A major aim of this short review was to assess the relationship of this genetic correlation with the extent of competitive stress exerted on the legume species, considering the available information for forage and grain legume species. Implications of the results on predicted genetic gains from selection in PS and MS, and complementary information on the relationships of cultivar competitive ability with total yield of the intercrop and on general mixing ability (GMA) and specific mixing ability (SMA) effects, were discussed to devise needs for and efficient procedures of legume breeding for intercropping.

## Comparing mixed stand *versus* pure stand selection based on predicted yield gains

Direct selection for yield in the target MS conditions could be compared with indirect selection for MS based on yield in PS in terms of yield gains predicted for each selection strategy. In particular, the predicted Relative Efficiency (*E_R_*) of indirect selection in PS relative to direct selection in MS can be estimated by the formula (Falconer, 1952; Annicchiarico et al., 2019) (Equation 1):

(1) $$E_{R}=r_{G}(h_{PS}/h_{MS})$$

where *h_PS_* and *h_MS_* stand for square root values of the broad-sense heritability (*h^2^*) for yield in PS and MS, respectively, and *r_G_* is the genetic correlation for genotype yield responses across MS and PS. Hence, breeding decisions depend not only on *r_G_* but also on differences in heritability between PS and MS selection environments (with selection in PS being favored by higher heritability relative to MS).

We carried out a literature review of field experiments on perennial legume-grass or annual legume-cereal mixtures in which legume genotypes were evaluated for yield under MS and PS conditions. We retained 13 data sets in which the legume species was represented by at least 14 genotypes. We chose this minimal threshold because of the large confidence intervals of *r_G_*, which, at *P* < 0.05, would require 12 degrees of freedom to exclude both zero and unity values for an estimate of *r_G_* = 0.5 according to the approximate formula in Windig (1997). **Table 1** provides *r_G_*, *h_PS_*, *h_MS_*, and $E_{R}$ values (when available) in these data sets. A few selected studies did not report *r_G_* values but provided estimates of other parameters that allowed us to estimate *r_G_*. In these cases, *r_G_* was estimated by the formula (Burdon, 1977; Basford et al., 2004) (Equation 2):

**Table 1:** Broad sense heritability (*h^2^*) and genetic correlation (*r_G_*) for yield of legume species in pure stand (PS) and mixed stand (MS), relative efficiency (*E_R_*) of indirect selection for yield in PS relative to direct selection in MS, and legume genotype mean yield ratio between MS and PS (*YR*), in legume intercropping studies.

Study	Focal species	Partner species	Sample size^a^	*h^2^* in PS	*h^2^* in MS	*r_G_* ^b^	*E_R_*	*YR* ^c^
Rowe and Brink (1993)	Trifolium repens	Cynodon dactylon (common)	47 G	0.72	0.35	0.53	0.76	0.40
Rowe and Brink (1993)	Trifolium repens	Cynodon dactylon (hybrid)	47 G	0.72	0.60	0.76	0.58	0.55
Annicchiarico (2003)	Trifolium repens	Lolium multiflorum + Festuca arundinacea	165 G	0.40	0.76	0.67	0.49	0.86
Annicchiarico (2003)	Trifolium repens	Lolium multiflorum + Festuca arundinacea	16 G	n.a.	n.a.	0.49	n.a.	0.45
Maamouri et al. (2017)	Medicago sativa	Festuca arundinacea	46 G	0.69	0.89	1.00	0.88	2.56
Atuahene-Amankwa and Michaels (1997)	Phaseolus vulgaris	Zea mays	16 FP	0.73	0.60	0.45	0.50	0.68
Zimmermann et al. (1984)	Phaseolus vulgaris	Zea mays	16 FP	0.21	0.23	0.52	0.50	0.18
Padi (2007)	Vigna unguiculata	Sorghum bicolor	24 G	0.80	0.49	*0.83*	1.07	1.26
Annicchiarico et al. (2021)	Pisum sativum	Hordeum vulgare + Triticum aestivum	144 G	0.71	0.66	0.43	0.44	0.38
Haug et al. (2023)	Pisum sativum	Hordeum vulgare	23 G	n.a.	0.37	*0.60*	n.a.	0.70
Sharma et al. (1993)	Glycine max	Zea mays	45 FP	n.a.	n.a.	0.52	n.a.	0.82
Sood and Sood (2001)	Glycine max	Zea mays	14 G	0.49	0.86	0.58	0.43	0.77
Atuahene-Amankwa et al. (2004)	Phaseolus vulgaris	Zea mays	16 G	0.70	0.65	0.40	0.42	0.58

^a^ For legume genotypes (G; as clones or inbred lines) or legume families or populations (FP).

^b^ Stated by authors or computed according to Equation 2; values in italics are phenotypic correlations across multi-environment trials considered as *r_G_* estimates.

^c^ Based on legume plant yield, or legume plot yield reported to same unit area.

(2) $$r_{G}=r_{P}/(h_{PS}\;h_{MS})$$

where *r_P_* is the phenotypic correlation for genotype yield responses across MS and PS, and *h_PS_* and *h_MS_* correspond to previous notations. In a few other cases, *r_G_* was not reported but we considered the phenotypic correlation between genotype yields in PS and genotype yields averaged over more than one MS environment as a reliable estimate of *r_G_*.

The *r_G_*values in **Table 1** averaged 0.60 but showed a wide range of variation (0.40-1.00). The average values of *h_PS_* and *h_MS_* (computed when both were available for a given data set) were nearly identical, i.e., 0.61 *versus* 0.60. As a result, the predicted efficiency of indirect selection in PS relative to direct selection in MS averaged 0.61 (implying nearly 40% lower predicted gains for indirect selection in PS), with wide variation (0.42-1.07) mainly determined by the variation of *r_G_* (**Table 1**). However, distinctly greater *h_PS_* than *h_MS_* occurred in the only data set featuring greater predicted efficiency of indirect selection in PS, the one of cowpea genotypes in association with sorghum by Padi (2007) (**Table 1**).

## Genotype consistency across pure stand and mixed stand depends on the extent of competitive stress

For each data set in **Table 1**, we expressed the extent of competitive stress exerted on the legume species as the yield ratio (*YR*) between mean yield in MS (*Y_MS_*) and mean yield in PS (*Y_PS_*) of the legume genotypes (Equation 3):

(3) $$YR=Y_{MS}/Y_{PS}$$

where *Y_MS_* and *Y_PS_* values were relative either to legume plant yield, or legume plot yield reported to same unit area (by doubling the values recorded in MS plots, unless they had been doubled already in the report). Hence, *YR* distinctly below one indicated high competitive stress experienced by the legume as a consequence of greater inter-specific competition encountered in MS relative to intra-specific competition in PS. *YR* values in **Table 1** averaged 0.78 with wide variation, which ranged from 0.18 for common bean in association with maize to 2.56 for alfalfa in association with tall fescue. A dominant legume showing relatively better mean yield response in MS than PS (*YR* above one) was observed only for alfalfa-tall fescue mixtures in Maamouri et al. (2017) and cowpea-sorghum mixtures in Padi (2007). These data sets featured the highest values of *r_G_* and $E_{R}$ in **Table 1**.

**Figure 1:**
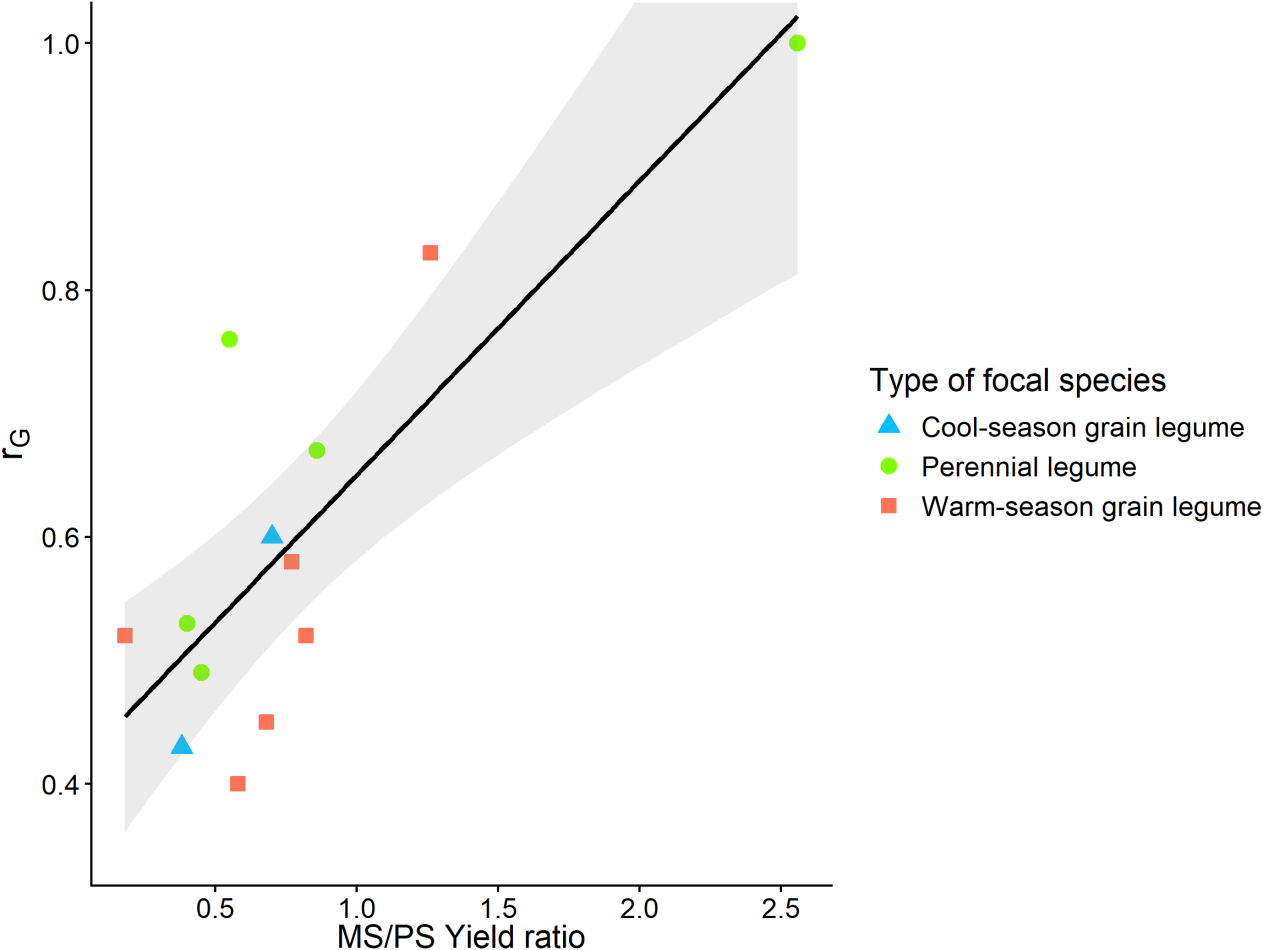
Linear regression of the genetic correlation (*r_G_*) for legume genotype yield across mixed stand (MS) and pure stand (PS) conditions as a function of the legume genotype mean yield ratio between MS and PS, for pooled data sets of different focal species (see **Table 1** for source data). Shaded areas represent 0.95 confidence intervals.

We assessed the presence of a relationship of *r_G_* across PS and MS conditions with *YR* for the pooled information of data sets reported in **Table 1** using the PROC REG of the SAS software and the ‘lm’ function of the R software. We found a significant (*P* < 0.001) positive linear relationship which accounted for 67% of the *r_G_* variation (*R^2^* = 0.67), with intercept of 0.41 and regression coefficient of 0.24. We found no significance for a curvilinear regression parameter (*P* > 0.30). The linear relationship is reported in **Figure 1**, which also shows the contributing data for three types of focal species (perennial legume; warm-season grain legume; cool-season grain legume). Performing this meta-analysis separately for each type of focal species was prevented by the small number of data sets.

The increase of legume genotype inconsistency across PS and MS environments with the increase of competitive stress exerted on the legume parallels what is usually observed for other stresses, e.g. drought, in which the genotype inconsistency relative to yield responses in moisture-favorable environments (i.e., absence of stress) increases with the increase of the drought stress level (Ceccarelli, 1996). The results in **Figure 1**, which are based on reports for different species, genotypes, and growing environments, agree with results of an experiment by Annicchiarico and Piano (1994) in which the legume material and any environmental factor were identical across several MS conditions. The experiment evaluated six white clover genotypes for biomass yield in PS and in binary mixture with each of eight grass cultivars belonging to four different species. The small number of clover genotypes prevented a meaningful estimation of *r_G_*across PS and each MS condition. However, the genotype inconsistency across PS and MS conditions increased just as a function of the competitive stress exerted on the clover regardless of the grass species, ranging from minimal in association with a poorly competing grass cultivar to substantial in mixtures with highly vigorous grass cultivars.

Grass and cereal species were less targeted by breeding research for intercropping than legume species, but a few studies suggest that the results reported in **Figure 1** may largely apply also to these types of species. A very low genetic correlation for genotype yield between PS and MS (*r_G_* = 0.25) was reported for 45 tall fescue half-sib families in association with a highly competing legume such as alfalfa (Waldron et al., 2017). In contrast, no genotype × environment interaction across PS and MS environments, and *r_G_* close to one (estimated from reported *r_P_* and heritability values), occurred for 31 sorghum cultivars that were dominating in associations with cowpea (Raboin et al., 2025).

Most legume species tend to be outcompeted in intercropping. This is usually the case for white clover, common bean, pea, and soybean according to results reported in **Table 1** and other studies for these species summarized in Bedoussac et al. (2015), Annicchiarico et al. (2019), or Harris (1987). A competitive disadvantage was frequently reported also for other cool-season grain legumes such lentil, white lupin, or vetches (Schmidtke et al., 2004; Mariotti et al., 2009; Annicchiarico et al., 2017), warm-season grain legumes such as cowpea, pigeon pea, and groundnut intercropped with maize or sorghum (Ofori and Stern, 1987), and red clover in MS with grass species (Brophy et al., 2017). Major exceptions to this trend are represented by the mostly good competitive ability of alfalfa with grasses (Maamouri et al., 2017; Annicchiarico and Pecetti, 2022; Surault et al., 2024) and faba bean with cereals (Xiao et al., 2018; Moutier et al., 2022; Kottelenberg et al., 2026).

## Legume competitive ability can affect total mixture yield

An asymmetrical competition leading to a competitive advantage of one species over another is frequent in any type of intercrop (Connell, 1983) and may strongly hamper the agroecologically efficient functioning of legume-based mixtures (Corre-Hellou et al., 2006). According to Harper’s (1977) general observation that the yield efficiency of a mixture depends mainly on the performance of its weaker partner, a definite competitive disadvantage of the legume component in a mixture is expected to impact negatively on the total yield of the intercrop, as confirmed by experimental (Zannone et al., 1986; Ofori and Stern, 1987) and modelling approaches (Schwinning and Parsons, 1996). The latter approaches showed that poor competitive ability of perennial legumes not only reduces the legume content but makes it more exposed to fluctuations (Schwinning and Parsons, 1996). As an alternative to selecting legume species for improved competitive ability, one may think of selecting non-legume companions for reduced competitive ability and/or adopting MS conditions that alleviate the competition exerted on the legume, such as the choice of less productive partner cultivars, very low or nil N fertilization, increased legume sowing density, and crop spatial arrangements that reduce the interspecific competition (Frame and Newbould, 1986; Ofori and Stern, 1987; Jensen et al., 2015; Kottelenberg et al., 2026). However, less productive partners (Zannone et al., 1986; Annicchiarico and Piano, 1994) or relatively greater legume sowing density (Yu et al., 2016; Lebreton et al., 2024) may decrease the total yield of the intercrop; very low or nil N fertilization may reduce the total yield and/or quality of the intercrop (Takele et al., 2017; Angeletti et al., 2024); and arrangements that reduces plant competition, such as strip cropping, also reduce the scope for exploiting functional diversity mechanisms. On the other hand, the selection for increased competitive ability of poorly-competing legume species tended to also increase the total yield of the intercrop for both perennial (Annicchiarico and Piano, 1994; Annicchiarico and Proietti, 2010) and annual legumes (Annicchiarico et al., 2021), owing to greater soil N available for plants of the non-legume component and, especially for perennials, more biologically-fixed nitrogen made available to the grass component (Oberson et al., 2013; Stomph et al., 2020). The importance of legume competitive ability is confirmed by its use as a key factor for estimating the total yield of wheat-grain legume intercrops (Kammoun et al., 2021). The whole of the above indications agrees with Zannone et al.’s (1986) conclusion, verified on Annicchiarico and Piano’s (1994) data in Annicchiarico et al. (2019), that total mixture yield tends to be maximized by partners with highest intrinsic yielding ability and lowest difference in competitive ability.

## Practical implications and additional considerations for legume species selection

Legume breeding for intercropping is challenged by possibly different companion species and many possible companion cultivars. In addition, the competition exerted by the partner species may vary largely as a function of N inputs, sowing density, and other crop management factors (Harris, 1987; Stomph et al., 2020; Moutier et al., 2022; Kottelenberg et al., 2026). The results from **Figure 1** and Annicchiarico and Piano (1994) can greatly simplify the challenge, suggesting that the expected level of competitive stress (as determined by highly productive partner genotypes and most likely crop management practices) is the key determinant for decisions on MS or PS selection environments and on MS selection conditions. Based on their indications, specific breeding for intercropping is indispensable for legume species expected to face high competition under the most likely conditions of mixed cropping. In that situation, legume selection for higher competitive ability may improve not only the quality (via increased legume content) but also the total yield of the intercrop. The selection could be carried out using just a few highly productive non-legume testers, which could conveniently be mixed in the same plot as in Annicchiarico (2003) to reduce selection costs. This selection procedure is supported by the reportedly modest size of SMA effects relative to GMA effects (Holland and Brummer, 1999; Maamouri et al., 2017; Haug et al., 2023), while results in Annicchiarico and Piano (1994) suggest that inconsistencies for legume genotype yield across different MS conditions may be substantial only if these conditions are highly contrasting for the extent of competitive stress exerted on the legume. The field-based selection workload in MS could also be reduced by using incomplete factorial designs (Haug et al., 2021).

Legume selection for intercropping may consider not only the yield of the legume genotype but also that of its associated species, i.e., the legume genotype’s indirect genetic effect (Bourke et al., 2021; Haug et al., 2023; Firmat and Litrico, 2022). For a poorly competing legume, however, indications discussed in the preceding section suggest that selection for greater competitive ability is a key determinant of GMA and total mixture yield, with a likely modest negative impact on the yield of the associated species. In this case, the costs of large-scale selection in MS may be reduced by focusing only on legume genotype yield rather than also on yield of the associated species – a decision possibly supported also by greater market or feed value of the legume species. Further savings of costs and time may arise from genome-enabled selection for legume yield in MS in place of field-based selection (Annicchiarico et al., 2021). Selecting a poorly competing legume only on the basis of legume yield would produce selection gains depending on traits associated to greater competitive ability, such as higher relative growth rate and taller plant stature (which are key under relatively favorable conditions to compete for light), as well as other traits associated to better spatial or temporal complementarity or plant plasticity of the legume (Annicchiarico et al., 2019). Some of these traits, if easily measurable in PS, offer the alternative strategy to select for MS by indirect selection in PS based on a selection index, with a predicted efficiency that may approach that of direct selection for yield in MS (Annicchiarico, 2003; Annicchiarico et al., 2021).

For a poorly-competing species such as white clover, a low-cost selection procedure based on clover yield in association with a mixture of two highly-productive grass cultivars of different species produced a new cultivar that maximized mixture total yields and featured about two-fold greater legume content than other cultivars under severe competition (Annicchiarico and Proietti, 2010). In this condition, the new cultivar reached the average clover content of 30% on an annual dry matter basis that is considered necessary to fully profit of the clover benefits in terms of crop quality and N fixation (Thomas, 1992).

Legume species featuring intrinsically high competitive ability could still be object of specific breeding for intercropping, but their genotype yield responses are expected to be largely consistent across PS and MS environments. This would support either the selection for specific traits conferring temporal and/or spatial niche complementarity (Litrico and Violle, 2015; Louarn et al., 2020; Demie et al., 2022; Moore et al., 2022) conveniently assessed in PS, or the selection in MS with a high focus also on indirect genetic effects leading to higher yield of the non-legume companion (Bourke et al., 2021; Haug et al., 2021; Firmat and Litrico, 2022).

While multi-species legume-based mixtures are receiving increasing attention (Bybee-Finley et al., 2023; Zetterlind et al., 2025), legume breeding for intercropping has been studied with respect to binary mixtures. However, results for white clover and red clover by Nyfeler et al. (2009) suggest that selecting poorly competitive legume species for higher competitive ability can be important also to raise the long-term yield and legume proportion of multi-species mixtures.

## References

[B1] Altieri M. A. (2004). Linking ecologists and traditional farmers in the search for sustainable agriculture. Front. Ecol. Environ. 2, 35–42. doi: 10.1890/1540-9295(2004)002[0035:LEATFI]2.0.CO;2

[B2] Angeletti F. G. S. Pampana S. Arduini I. Saia S. Mariotti M. (2024). Can nitrogen fertilization and intercropping modify the quality and nutrient yield of barley–field bean forage? Agronomy 14, 1166. doi: 10.3390/agronomy14061166 30654563

[B3] Annicchiarico P. (2003). Breeding white clover for increased ability to compete with associated grasses. J. Agr. Sci. 140, 255–266. doi: 10.1017/S0021859603003198 41292463

[B4] Annicchiarico P. Collins R. P. De Ron A. M. Firmat C. Litrico I. Hauggaard-Nielsen H. (2019). Do we need specific breeding for legume-based mixtures? Adv. Agron. 157, 141–215. doi: 10.1016/bs.agron.2019.04.001

[B5] Annicchiarico P. Nazzicari N. Notario T. Monterrubio Martin C. Romani M. Ferrari B. . (2021). Pea breeding for intercropping with cereals: variation for competitive ability and associated traits, and assessment of phenotypic and genomic selection strategies. Front. Plant Sci. 12, 731949. doi: 10.3389/fpls.2021.731949 34630481 PMC8495324

[B6] Annicchiarico P. Pecetti L. (2022). Exploiting heterosis of semi-hybrids and heterogeneity of cultivar mixtures to enhance alfalfa crop performance. Field Crops Res. 283, 108522. doi: 10.1016/j.fcr.2022.108522 38826717

[B7] Annicchiarico P. Piano E. (1994). Interference effects in white clover genotypes grown as pure stands and binary mixtures with different grass species and varieties. Theor. Appl. Genet. 88, 153–158. doi: 10.1007/BF00225891 24185920

[B8] Annicchiarico P. Proietti S. (2010). White clover selected for enhanced competitive ability widens the compatibility with grasses and favours the optimization of legume content and forage yield in mown clover-grass mixtures. Grass Forage Sci. 65, 318–324. doi: 10.1111/j.1365-2494.2010.00749.x

[B9] Annicchiarico P. Thami Alami I. Abbas K. Pecetti L. Melis R. A. M. Porqueddu C. (2017). Performance of legume-based annual forage crops in three semi-arid Mediterranean environments. Crop Pasture Sci. 68, 932–941. doi: 10.1071/CP17068

[B10] Atuahene-Amankwa G. Michaels T. E. (1997). Genetic variances, heritabilities and genetic correlations of grain yield, harvest index and yield components for common bean (*Phaseolus vulgaris* L.) in sole crop and in maize/bean intercrop. Can. J. Plant Sci. 77, 533–538. doi: 10.4141/P96-168

[B11] Atuahene-Amankwa G. Beatie A. D. Michaels T. E. Falk D. E. (2004). Cropping system evaluation and selection of common bean genotypes for a maize/bean intercrop. Afr. Crop Sci. J. 12, 105–113. doi: 10.4314/acsj.v12i2.27668

[B12] Basford K. E. Federer W. T. DeLacy I. H. (2004). Mixed model formulations for multi-environment trials. Agron. J. 96, 143–147. doi: 10.2134/agronj2004.1430

[B13] Bedoussac L. Journet E. Hauggaard-Nielsen H. Naudin C. Corre Hellou G. Jensen E. . (2015). Ecological principles undelying the increase of productivity achieved by cereal-grain legume intercrops in organic farming. Agron. Sustain. Dev. 35, 911–935. doi: 10.1007/s13593-014-0277-7 30311153

[B14] Bourke P. M. Evers J. B. Bijma P. van Apeldoorn D. F. Smulders M. J. M. Kuyper T. W. . (2021). Breeding beyond monoculture: putting the “intercrop” into crops. Front. Plant Sci. 12, 734167. doi: 10.3389/fpls.2021.734167 34868116 PMC8636715

[B15] Brooker R. W. Bennett A. E. Cong W. Daniell T. J. George T. S. Hallett P. D. (2015). Improving intercropping: a synthesis of research in agronomy, plant physiology and ecology. New Phytol. 206, 107–117. doi: 10.1111/nph.13132 25866856

[B16] Brooker R. W. Pakeman R. J. Adam E. . (2024). Positive effects of intercrop yields in farms from across Europe depend on rainfall, crop composition, and management. Agron. Sustain. Dev. 44, 35–117. doi: 10.1007/s13593-024-00968-2 25866856

[B17] Brophy C. Finn J. A. Lüscher A. Suter M. Kirwan L. Sebastià M. T. . (2017). Major shifts in species’ relative abundance in grassland mixtures alongside positive effects of species diversity in yield: a continental‐scale experiment. J. Ecol. 105, 1210–1222. doi: 10.1111/1365-2745.12754 40046247

[B18] Burdon R. D. (1977). Genetic correlation as a concept for studying genotype-environment interaction in forest tree breeding. Silvae Genet. 26, 168–175.

[B19] Bybee-Finley K. A. Menalled U. D. Pelzer C. J. Ruhl L. Lounsbury N. P. Warren N. D. . (2023). Quantifying the roles of intraspecific and interspecific diversification strategies in forage cropping systems. Field Crops Res. 302, 109036. doi: 10.1016/j.fcr.2023.109036 38826717

[B20] Ceccarelli S. (1996). Adaptation to low/high input cultivation. Euphytica 92, 203–214. doi: 10.1007/BF00022846 30311153

[B21] Connell J. H. (1983). On the prevalence and relative importance of interspecific competition: evidence from field experiments. Am. Nat. 122, 661–636. doi: 10.1086/284165 24784085

[B22] Corre-Hellou G. Fustec J. Crozat Y. (2006). Interspecific competition for soil N and its interaction with N2 fixation, leaf expansion and crop growth in pea-barley intercrops. Plant Soil 282, 195–208. doi: 10.1007/s11104-005-5777-4 30311153

[B23] Demie D. T. Döring T. F. Finckh M. R. van der Werf W. Enjalbert J. Seidel S. J. (2022). Mixture × genotype effects in cereal/legume intercropping. Front. Plant Sci. 13, 846720. doi: 10.3389/fpls.2022.846720 35432405 PMC9011192

[B24] Dubey R. Zustovi R. Landschoot S. Dewitte K. Verlinden G. Haesaert G. . (2024). Harnessing monocrop breeding strategies for intercrops. Front. Plant Sci. 15, 1394413. doi: 10.3389/fpls.2024.1394413 38799097 PMC11119317

[B25] Falconer D. S. (1952). The problem of environment and selection. Am. Nat. 86, 293–298. doi: 10.1086/281736 33376296

[B26] Firmat C. Litrico I. (2022). Linking quantitative genetics with community-level performance: are there operational models for plant breeding? Front. Plant Sci. 13, 733996. doi: 10.3389/fpls.2022.733996 36340376 PMC9627035

[B27] Frame J. Newbould P. (1986). Agronomy of white clover. Adv. Agron. 40, 1–88. doi: 10.1016/s0065-2113(08)60280-1

[B28] Harper J. L. (1977). Population biology of plants (London: Academic Press).

[B29] Harris W. (1987). “ Population dynamics and competition,” in White clover. Eds. Baker M. J. Williams W. M. ( CABI, Wallingford, UK), 203–297.

[B30] Haug B. Messmer M. M. Enjalbert J. Goldringer I. Flutre T. Mary-Huard T. . (2023). New insights towards breeding for mixed cropping of spring pea and barley to increase yield and yield stability. Field Crops Res. 297, 108923. doi: 10.1016/j.fcr.2023.108923 38826717

[B31] Haug B. Messmer M. Enjalbert J. Goldringer I. Forst E. Flutre T. . (2021). Advances in breeding for mixed cropping - incomplete factorials and the producer/associate concept. Front. Plant Sci. 11, 620400. doi: 10.3389/fpls.2020.620400 33505418 PMC7829252

[B32] Hohmann P. Kussmann S. Schöb C. Rubiales D. Annicchiarico P. (2026). Rethinking variety testing to recognise mixing ability: bridging breeding, policy, and practice in diversified agriculture. Front. Plant Sci. 17, 1770790. doi: 10.3389/fpls.2026.1770790 41929838 PMC13038573

[B33] Holland J. B. Brummer E. C. (1999). Cultivar effects on oat-berseem clover intercrops. Agron. J. 91, 321–329. doi: 10.2134/agronj1999.00021962009100020023x

[B34] Jensen E. S. Bedoussac L. Carlsson G. Journet E. P. Justes E. Hauggaard-Nielsen H. (2015). Enhancing yields in organic arable crop production by eco-functional intensification using intercropping. Sustain. Agr. Res. 4, 3–8. doi: 10.5539/sar.v4n3p42

[B35] Justes E. Bedoussac L. Dordas C. Frak E. Louarn G. Boudsocq S. . (2021). The 4C approach as a way to understand species interactions determining intercropping productivity. Front. Agr. Sci. Eng. 8, 387–399. doi: 10.15302/J-FASE-2021414

[B36] Kammoun B. Journet E.-P. Justes E. Bedoussac L. (2021). Cultivar grain yield in durum wheat-grain legume intercrops could be estimated from sole crop yields and interspecific interaction index. Front. Plant Sci. 12, 733705. doi: 10.3389/fpls.2021.733705 34721461 PMC8551613

[B37] Kiær L. P. Skovgaard I. M. Østergård H. (2009). Grain yield increase in cereal variety mixtures: a meta-analysis of field trials. Field Crops Res. 114, 361–373. doi: 10.1016/j.fcr.2009.09.006 38826717

[B38] Kiær L. P. Weedon O. D. Bedoussac L. Bickler C. Finckh M. R. Haug B. . (2022). Supply chain perspectives on breeding for legume-cereal intercrops. Front. Plant. Sci. 13, 844635. doi: 10.3389/fpls.2022.844635 35300006 PMC8921979

[B39] Kottelenberg D. B. Evers J. B. Anten N. P. R. Bastiaans L. (2026). Managing species dominance in cereal-legume intercrop systems. Eur. J. Agron. 177, 128065. doi: 10.1016/j.eja.2026.128065 38826717

[B40] Lebreton P. Bedoussac L. Bonnet C. Journet E.-P. Justes E. Colbach N. (2024). Optimal species proportions, traits and sowing patterns for agroecological weed management in legume–cereal intercrops. Eur. J. Agron. 159, 127266. doi: 10.1016/j.eja.2024.127266 38826717

[B41] Li C. Stomph T. J. Makowski D. Li H. Zhang C. Zhang F. . (2023). The productive performance of intercropping. Proc. Natl. Acad. Sci. 120, 2201886120. doi: 10.1073/pnas.2201886120 36595678 PMC9926256

[B42] Litrico I. Violle C. (2015). Diversity in plant breeding: a new conceptual framework. Trends Plant Sci. 20, 604–613. doi: 10.1016/j.tplants.2015.07.007 26440430

[B43] Louarn G. Barillot R. Combes D. Escobar-Gutiérrez A. (2020). Towards intercrop ideotypes: non-random trait assembly can promote overyielding and stability of species proportion in simulated legume-based mixtures. Ann. Bot. 126, 671–685. doi: 10.1093/aob/mcaa014 32004372 PMC7489071

[B44] Maamouri A. Louran G. Béguier V. Julier B. (2017). Performance of lucerne genotypes for biomass production and nitrogen content differs in monoculture and in mixture with grasses and is partly predicted from traits recorded on isolated plants. Crop Pasture Sci. 68.11, 942–951. doi: 10.1071/CP17052 38477348

[B45] Malleson C. (2016). Informal intercropping of legumes with cereals? A re-assessment of clover abundance in ancient Egyptian cereal processing by-product assemblages: archeobotanical investigations at Khentkawes town, Giza, (2300-2100 BC). Veget. Hist. Archaeobot. 25, 431–442. doi: 10.1007/s00334-016-0559-x 30311153

[B46] Mariotti M. Masoni A. Ercoli L. Arduini I. (2009). Above- and below-ground competition between barley, wheat, lupin and vetch in a cereal and legume intercropping system. Grass Forage Sci. 64, 401–412. doi: 10.1111/j.1365-2494.2009.00705.x 40046247

[B47] Moore V. M. Schlautman B. Fei S. Roberts L. M. Wolfe M. Ryan M. R. . (2022). Plant breeding for intercropping in temperate field crop systems: a review. Front. Plant Sci. 13, 843065. doi: 10.3389/fpls.2022.843065 35432391 PMC9009171

[B48] Mota-Cruz C. Casas A. Ortega-Paczka R. Perales H. Vega-Peña E. Bye R. (2025). Milpa, a long-standing polyculture for sustainable agriculture. Agriculture 15, 1737. doi: 10.3390/agriculture15161737 30654563

[B49] Moutier N. Baranger A. Fall S. Hanocq E. Marget P. Floriot M. . (2022). Mixing ability of intercropped wheat varieties: stability across environments and tester legume species. Front. Plant Sci. 13, 877791. doi: 10.3389/fpls.2022.877791 35755684 PMC9218859

[B50] Nyfeler D. Huguenin-Elie O. Suter M. Frossard E. Connolly J. Lüscher A. (2009). Strong mixture effects among four species in fertilized agricultural grassland led to persistent and consistent transgressive overyielding. J. Appl. Ecol. 46, 683–691. doi: 10.1111/j.1365-2664.2009.01653.x 40046247

[B51] Oberson A. Frossard E. Bühlmann C. Mayer J. Mäder P. Lüscher A. (2013). Nitrogen fixation and transfer in grass-clover leys under organic and conventional cropping systems. Plant Soil 371, 237–255. doi: 10.1007/s11104-013-1666-4 30311153

[B52] Ofori F. Stern W. R. (1987). Cereal-legume intercropping systems. Adv. Agron. 41, 41–90. doi: 10.1016/S0065-2113(08)60802-0

[B53] Padi F. K. (2007). Genotype × environment interaction and yield stability in a cowpea-based cropping system. Euphytica 158, 11–25. doi: 10.1007/s10681-007-9420-8 30311153

[B54] Raboin L. M. Ouedraogo N. Kabore M. Zabre Y. Ganeme A. Bonnal L. . (2025). Sorghum genetic variability for adaptation to intercropping with cowpea in Sudano-Sahelian conditions. Field Crops Res. 330, 109984. doi: 10.1016/j.fcr.2025.109984 38826717

[B55] Rowe D. E. Brink G. E. (1993). Heritabilities and genetic correlations of white clover clones grown in three environments. Crop Sci. 33, 1149–1152. doi: 10.2135/cropsci1993.0011183X003300060008x

[B56] Schmidtke K. Neumann A. Hof C. Rauber R. (2004). Soil and atmospheric nitrogen uptake by lentil (*Lens culinaris* Medik.) and barley (*Hordeum vulgare* ssp. *nudum* L.) as monocrops and intercrops. Field Crops Res. 87, 245–256. doi: 10.1016/j.fcr.2003.11.006 38826717

[B57] Schneider A. Huyghe C. Maleplate T. Labalette F. Peyronnet C. Carrouée B. (2015). “ Rôle des légumineuses dans l’agriculture française,” in Les légumineuses pour des systèmes agricoles et alimentaires durables. Eds. Schneider A. Huyghe C. ( Editions Quae, Versailles, France), 11–77.

[B58] Schwinning S. Parsons A. J. (1996). Analysis of the coexistence mechanisms for grasses and legumes in grazing systems. J. Ecol. 84, 799–813. doi: 10.2307/2960553

[B59] Sharma S. K. Mehta H. Sood V. K. (1993). Effect of cropping systems on combining ability and gene action for grain yield and its components in soybean. Field Crops Res. 34, 15–22. doi: 10.1016/0378-4290(93)90107-X

[B60] Sood V. K. Sood O. P. (2001). Effect of cropping system on some genetic parameters in soybean. Indian J. Genet. Plant Breed. 61, 132–135.

[B61] Stomph T. Dordas C. Baranger A. de Rijk J. Dong B. Evers J. . (2020). Designing intercrops for high yield, yield stability and efficient use of resources: Are there principles? Adv. Agron. 160, 1–50. doi: 10.1016/bs.agron.2019.10.002 38826717

[B62] Surault F. Huyghe C. Sampoux J.-P. Larbre D. Barre P. Louarn G. . (2024). Weed control, protein and forage yield of seven grass species in lucerne-grass associations. Field Crops Res. 309, 109308. doi: 10.1016/j.fcr.2024.109308 38826717

[B63] Takele E. Mekonnen Z. Tsegaye D. Abebe A. (2017). Effect of intercropping of legumes and rates of nitrogen fertilizer on yield and yield components of maize (*Zea mays* L.) at Arba Minch. Am. J. Plant Sci. 8, 2159–2179. doi: 10.4236/ajps.2017.89145

[B64] Tamburini G. Bommarco R. Wanger T. C. Kremen C. van der Heijden M. G. A. Liebman M. . (2020). Agricultural diversification promotes multiple ecosystem services without compromising yield. Sci. Adv. 6, eaba1715. doi: 10.1126/sciadv.aba1715 33148637 PMC7673676

[B65] Thomas R. J. (1992). The role of legumes in the nitrogen cycle of productive and sustainable pastures. Grass Forage Sci. 47, 133–142. doi: 10.1111/j.1365-2494.1992.tb02256.x 40046247

[B66] Waldron B. L. Peel M. D. Larson S. R. Mott I. W. Creech J. E. (2017). Tall fescue forage mass in a grass-legume mixture: predicted efficiency of indirect selection. Euphytica 213, 67. doi: 10.1007/s10681-017-1856-x 30311153

[B67] Wang W. Li M. Y. Wang Y. Li J. M. Zhang W. Wen Q. H. . (2025). Legume intercropping improves soil organic carbon stability in drylands: a 7-year experimental validation. Agric. Ecosyst. Environ. 381, 109456. doi: 10.1016/j.agee.2024.109456 38826717

[B68] Windig J. J. (1997). The calculation and significance testing of genetic correlations across environments. J. Evol. Biol. 10, 853–874. doi: 10.1111/j.1420-9101.1997.tb00002.x 40046247

[B69] Xiao J. Yin X. Ren J. Zhang M. Tang L. Zheng Y. (2018). Complementation drives higher growth rate and yield of wheat and saves nitrogen fertilizer in wheat and faba bean intercropping. Field Crops Res. 221, 119–129. doi: 10.1016/j.fcr.2017.12.009 38826717

[B70] Yu Y. Stomph T. J. Makowski D. van der Werf W. (2015). Temporal niche differentiation increases the land equivalent ratio of annual intercrops: a meta-analysis. Field Crop Res. 184, 133–144. doi: 10.1016/j.fcr.2015.09.010 38826717

[B71] Yu Y. Stomph T. J. Makowski D. Zhang L. Van der Werf W. (2016). A meta-analysis of relative crop yields in cereal/legume mixtures suggests options for management. Field Crop Res. 198, 269–279. doi: 10.1016/j.fcr.2016.08.001 38826717

[B72] Zannone L. Rotili P. Paoletti R. Scotti C. (1986). Experimental studies of grass-legume associations. Agronomie 6, 931–940. doi: 10.1051/agro:19861009 42236926

[B73] Zetterlind B. Janssen P. Geerts R. Visser T. Stip A. Zwetsloot M. J. . (2025). Multispecies grasslands sustain on-farm forage productivity and quality while reducing nitrogen fertilizer inputs. Plant Soil. doi: 10.1007/s11104-025-08006-0 30311153

[B74] Zimmermann M. J. Rosielle A. A. Waines J. G. Foster K. W. (1984). A heritability and correlation study of grain yield, yield components, and harvest index of common bean in sole crop and intercrop. Field Crops Res. 9, 109–118. doi: 10.1016/0378-4290(84)90017-0

